# Use of antibiotics for urinary tract infections up to and after care home admission in Denmark: a nationwide study

**DOI:** 10.1007/s41999-024-00976-1

**Published:** 2024-05-02

**Authors:** Emma Bjørk, Rune Aabenhus, Søren P. Larsen, Jesper Ryg, Daniel P. Henriksen, Carina Lundby, Anton Pottegård

**Affiliations:** 1 Hospital Pharmacy Funen, Odense University Hospital, Odense C, Denmark; 2 Department of Public Health, Clinical Pharmacology, Pharmacy and Environmental Medicine, University of Southern Denmark, JB Winsløwsvej 19, 2, 5000 Odense C, Denmark; 3 Odense Deprescribing Initiative (ODIN), Odense University Hospital, University of Southern Denmark, Odense C, Denmark; 4 Section of General Practice and Research Unit for General Practice, Department of Public Health, University of Copenhagen, Copenhagen, Denmark; 5 REAGENS, Skårup Fyn, Denmark; 6 Department of Geriatric Medicine, Odense University Hospital, Odense C, Denmark; 7 Geriatric Research Unit, Department of Clinical Research, University of Southern Denmark, Odense C, Denmark; 8 Department of Clinical Pharmacology, Odense University Hospital, Odense C, Denmark; 9 Department of Public Health, Research Unit of General Practice, University of Southern, Odense C, Denmark

**Keywords:** Care home, Drug utilization, Urinary tract infection, Antibiotics, Older people

## Abstract

**Purpose:**

Older people have the highest use of antibiotics for acute and chronic urinary tract infection (UTI), despite diagnostic uncertainty and the growing problem of antibiotic resistance. We aim to describe use-patterns of UTI antibiotics two years prior to and following care home admission in Denmark.

**Methods:**

This was a register-based nationwide drug-utilization study. In a cohort comprising all Danish residents admitted into a care home from 2015 to 2021, we described the use of UTI antibiotics, and examined differences between regions and individual care homes in rates of UTI antibiotic use. Further, we described trends in UTI-related contacts with hospitals in the two years prior to and following care home admission.

**Results:**

The cohort comprised 101,297 residents (61% female; median age 84 years). UTI antibiotic use doubled from 7 to 14 treatments/100 residents/month two months prior to care home admission and remained at 10 treatments/100 residents/month the following two years. Prescription of pivmecillinam (55%) was most common. Primary care practitioners prescribed the majority (92%) of UTI antibiotics. UTI-related hospital contacts peaked at two months prior to care home admission, with 6 admissions/100 residents/month, subsequently dropping to 2 admission/100 residents/month. We found considerable variation in UTI antibiotic use, with 10% of care homes responsible for 20% of treatments in 2021.

**Conclusion:**

Use of UTI antibiotics increased prior to and remained at a stable high level following care home admission in Denmark. Despite variation in use across regions and individual care homes, an overall decrease was seen throughout the years 2016–2021.

**Supplementary Information:**

The online version contains supplementary material available at 10.1007/s41999-024-00976-1.

## Key summary points

**Aim:**

To describe use-patterns of UTI antibiotics two years prior to and following care home admission in Denmark.

**Findings:**

Use of antibiotics for urinary tract infections double about six months prior to care home admission. Following care home admission, the use of antibiotics for urinary tract infection remains at a persistent high level.

**Message:**

Use of antibiotics for urinary tract infections shows an overall decrease throughout the years 2016 to 2021, despite variation between residents, care homes, and geographical regions.

**Supplementary Information:**

The online version contains supplementary material available at 10.1007/s41999-024-00976-1.

## Introduction

Antibiotic resistance is considered one of the biggest threats to global health by the World Health Organization (WHO) [1], among other things driven by a high use of antibiotics among older people [2–4]. Residents in care homes are vulnerable to infections [5–8] due to several factors, including immunosenescence [9], multimorbidity [6], use of catheters and feeding tubes [10–12], close living proximity [11, 12], and repeated and continuous contact with nursing staff and medical equipment [11, 12]. A European study on use of antimicrobial medications in long-term care facilities [13] showed that the most common type of antimicrobials prescribed were for urinary tract infections (UTIs) (46%), including both acute treatment episodes and long-term prophylaxis [13]. High use of UTI antibiotics in care homes may be driven by several factors, in particular a high prevalence of asymptomatic bacteriuria among such residents (up to 50%) compared to older people living in the community (4%), despite no reported benefit from antibiotic treatment for asymptomatic bacteriuria [14, 15]. Another contributing factor is the clinical uncertainty around common occurrences such as confusion, falls, and agitation being attributed to UTIs [16, 17]. The time after transitioning from independent living to living in care home is of particular interest, as this may precipitate behavioral changes such as increased confusion, irritability, and agitation often associated with care home admission [18, 19], in turn, possibly exposing the newly admitted residents to antibiotic treatments for presumed UTIs. The aim of this study is therefore to provide knowledge to help inform clinical guidelines and regulations by describing and characterizing patterns of antibiotic use for UTIs and UTI-related hospital contacts up to and after care home admission in a nation-wide cohort of Danish care home residents.

## Methods

This was a register-based national drug utilization study among a cohort of all people admitted into care homes across Denmark from 2015 to 2021. The cohort was linked with individual-level registry data on prescriptions filled in community pharmacies in Denmark.

### Design and data sources

The national cohort was assembled, encrypted, and provided by the Danish Health Data Authority using data from various Danish health registries and linked via the personal identification number assigned to all Danish residents since 1968 [20]. Individual-level data on filled prescriptions were collected from the Danish National Prescription Register [21], containing data on all prescriptions filled in community pharmacies in Denmark since 1995. This includes variables such as the type of drug, amount, and Anatomic Therapeutic Chemical (ATC) classification [22]. Data on hospital contacts and admissions were collected from the Danish National Patient Registry [23]. The Patient Registry includes variables such as admission and discharge diagnoses, coded using ICD-10 since 1994, hospital departments, and admission/discharge dates and times. Prescriber information from the Prescription Registry was linked with the Registry of Health Care Providers [24] to identify type of prescriber (primary health care sector versus secondary health care sector). Currently no national guideline exists on the selection of antibiotics for UTIs. However, regional guidelines primarily suggest pivmecillinam (J01CA08) as first-line treatment for UTIs [25–30]. The chosen antibiotics are in Danish clinical context used almost exclusively for UTIs [31–34], and was based on discussion with clinicians and on Danish Health Care Guidelines [25–30]. We examined the following oral antibiotics that are commonly used for UTI in Denmark: pivmecillinam (J01CA08), nitrofurantoin (J01XE01), trimethoprim (J01EA01), sulfonamide drugs (sulfamethizole (J01EB02) and sulfamethoxazole/trimethoprim (J01EE01)), and ciprofloxacin (J01MA02). UTI-related hospital contacts within the cohort was examined using the following diagnoses according to the Patient Registry [23]: cystitis (ICD-10: N30.X and N39.0), pyelonephritis (ICD-10: N10.X, N11.X and N12.X), observation due to suspected UTI (ICD-10: Z038A and Z038B), and urosepsis (ICD-10: A419B).

### Research questions

We structured the analyses across five individual research questions. All analyses were carried out both overall and stratified by sex. A treatment episode was defined by the filling of a prescription for a UTI antibiotic. Multiple fills within 15 days were considered to belong to the same treatment episode. As the data material included individuals moving into care homes from 2015 onwards, analyses of total use were restricted to 2016 onwards to ensure comparability in the cohorts over time. All analyses utilized the Danish National Prescription Register [21], while the fourth and fifth analysis, respectively, also utilized the Danish National Patient Registry [23], and the Registry of Health Care Providers [24].

First, to describe the overall rate of use of UTI antibiotics among care home residents, we calculated the total number of treatment episodes per month per 100 residents in the two years leading up to and following care home admission and specified by type of antibiotic.

Second, to describe differences in use of UTI antibiotics across the five Danish regions and 98 individual municipalities, as well as changes in the period 2016–2021, we calculated the annual average number of treatment episodes per person-year per location.

Third, to describe the skewness of use of UTI antibiotics among individual care homes, we generated an inverse Lorenz curve for the year 2021, restricting to care homes with more than 10 person-years follow-up among its residents. In short, a Lorenz curve [35] depicts the proportion of all drug use, here UTI antibiotics, used by a proportion of the population, here individual care homes. If the use of UTI antibiotics were equally distributed between care homes the Lorenz curve would thus be a straight line.

Fourth, to describe the trends of UTI-related hospital contacts within the cohort, we calculated number of contacts related to UTIs in the two years leading up to and following care home admission. Hospital contacts were restricted to primary diagnoses associated with emergency room admissions and inpatient admissions (thus excluding ambulatory contacts).

Lastly, we determined what type of doctors (primary health care sector; general practitioner and other specialists, secondary health care sector; hospital physician, unknown) are responsible for prescribing UTI antibiotics in the two years leading up to and following care home admission.

### Ethics and approvals

This study did not require approval from an ethics review board, according to Danish law on studies based solely on register data [36]. In terms of data protection, the study was registered at the repository of University of Southern Denmark (11.277).

## Results

The cohort comprised 101,297 residents, of which 61% were female and the median age was 84 years (Table 1). Most (78%) had at least one hospital contact (any cause) during the last six months prior to being admitted into a care home. One fifth of residents (n = 19,615) had passed away or were censored six months following care home admission. This increased to 36% (n = 36,024) and 60% (n = 60,539) one year and two years following care home admission, respectively.

**Table 1:** Baseline characteristics of Danish care home residents admitted from 2015 to 2021

	Total population (n = 101,297)	Before admission to care home	
		No UTI antibiotic use (n = 63,298) 62%	Antibiotic use (n = 37,999) 38%
Female, n (%)	61,598 (61)	35,459 (58)	26,139 (42)
Age, median (IQR)	84 (77–89)	83 (76–89)	85 (79–90)
Age, n (%)			
< 65	5,353 (5.3)	4,049 (76)	1,304 (24)
66–79	27,082 (27)	18,154 (67)	8,928 (33)
≥ 80	68,862 (68)	41,095 (60)	27,767 (40)
Hospital contacts during the six months prior to care home admission, n (%)			
0	22,506 (22)	17,178 (76)	5,328 (24)
1–2	50,344 (50)	30,479 (61)	19,865 (39)
≥ 3	28,447 (28)	15,641 (55)	12,806 (45)
Charlson Comorbidity Index, median (IQR)	2 (0–2)	2 (0–2)	2 (0–3)
Charlson Comorbidity Index, n (%)			
0	33,130 (33)	20,551 (62)	12,579 (38)
1	9,829 (9.7)	5,828 (59)	4,001 (41)
2	33,960 (34)	22,139 (65)	11,821 (35)
≥ 3	24,378 (24)	14,780 (61)	9,598 (39)

Overall and stratified for non-users and users of urinary tract infections antibiotics in the 180 days before admission to care home

*UTI* Urinary tract infection, *IQR* Interquartile range

In the year leading up to care home admission, a total of 45,522 individuals (45%) were prescribed at least one UTI antibiotic. Similarly, in the year following care home admission, there were 44,964 individuals (44%) filling UTI antibiotics. Among those using UTI antibiotics prior to care home admission, 22% also filled a prescription in the six months after care home admission. We identified a total of 200,877 and 222,844 prescriptions of UTI antibiotics in the two years prior to and following care home admission, respectively. Use of pivmecillinam (55%) was most common, followed by trimethoprim (18%), sulfonamides (10%), nitrofurantoin (9.1%), and ciprofloxacin (8.2%) (Supplementary Fig. 1, Supplementary Table 1). We found that 15% of all UTI antibiotic prescriptions were followed by another UTI antibiotic prescription within 15 days, most often a new prescription for the same antibiotic (51–60%), except for sulfonamides that were followed equally by either a new sulfonamide or pivmeciliam prescription (Supplementary Table 2). The 90-day mortality following care home admission was 14% and 11%, for residents receiving a UTI antibiotic within six months prior to admission and those without such a prescription, respectively. Similarly, the one-year mortality was 35% and 29%.

**Fig. 1:**
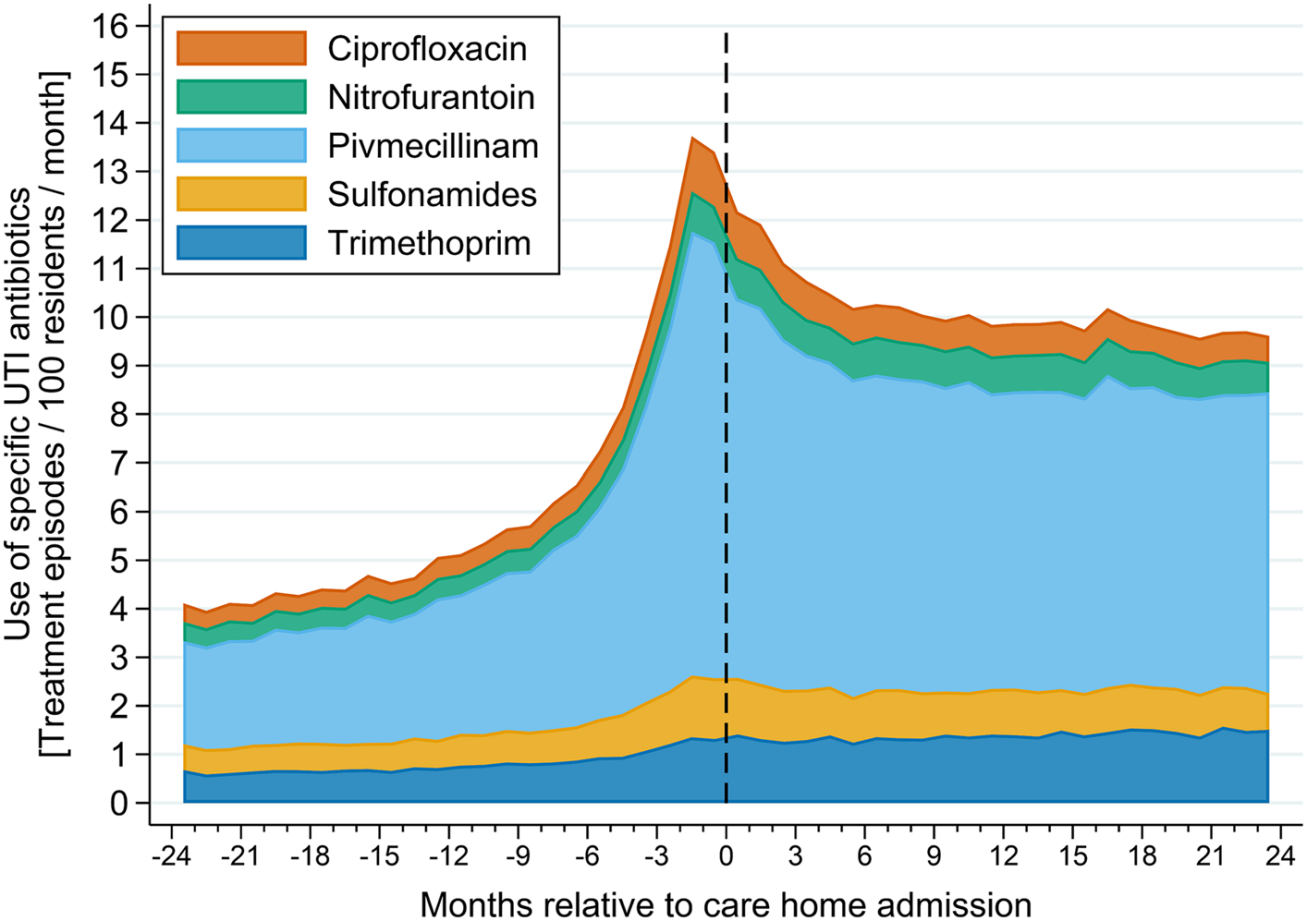
Number of treatment episodes per month per 100 residents in the 2 years leading up to and following care home admission and specified by type of antibiotic. A treatment episode was defined as the filling of a prescription for a urinary tract infection specific antibiotic ≥ 15 days after a previous prescription

Use of UTI antibiotics increased from around six months prior to care home admission from 7 treatment episodes per 100 residents per month to 14 treatment episodes per 100 residents per month at two months prior to care home admission (Fig. 1).

**Fig. 2:**
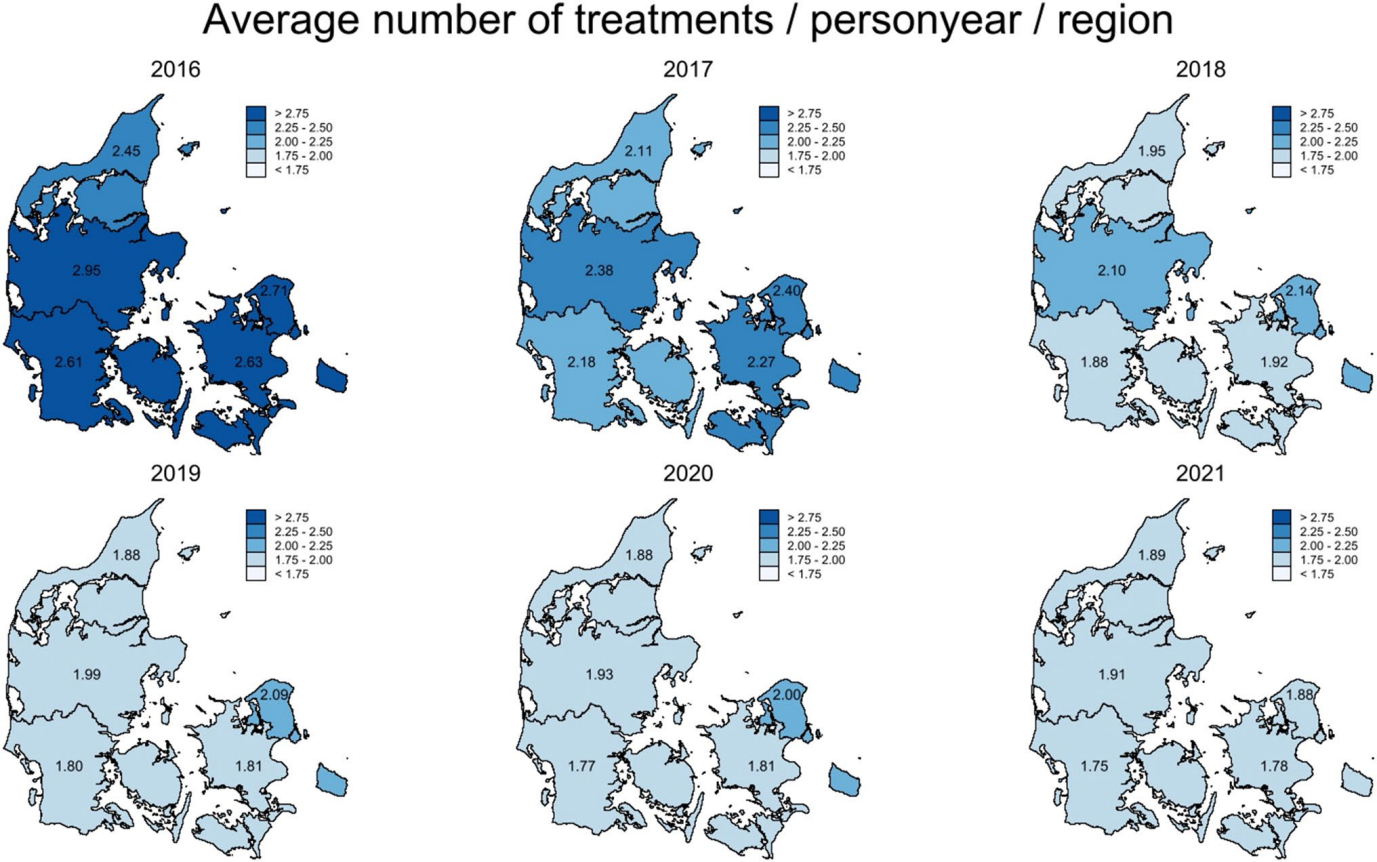
Differences in use of urinary tract infections antibiotics between the five regions of Denmark, during 2016 to 2021. Represented by average number of treatments per person-year per region, standardized by sex and age to the overall population. A treatment episode was defined as the filling of a prescription for a UTI antibiotic ≥ 15 days after a previous prescription

Following care home admission, this changed to 10 treatment episodes per 100 resident per months and stayed consistent for the following 2 years. The rate of UTI treatments was higher among women with a peak of 16 treatment episode per 100 residents per month two months prior to admission compared to that of men (11 treatment episodes per 100 residents per month two months prior to admission) (Supplementary Fig. 2). The distribution in use of UTI antibiotics were similar between men and women (Supplementary Fig. 3). The overall average number of treatment episodes per person-year in the five regions of Denmark decreased during the years from 2016 to 2021, ranging from 2.95 treatment episodes/person-year in 2016 to 1.75 treatment episodes/person-year in 2021 (Fig. 2). A similar trend of overall decrease in number of treatment episodes per person-year, was seen among the 98 different municipalities in Denmark (Supplementary Fig. 4).

**Fig. 3:**
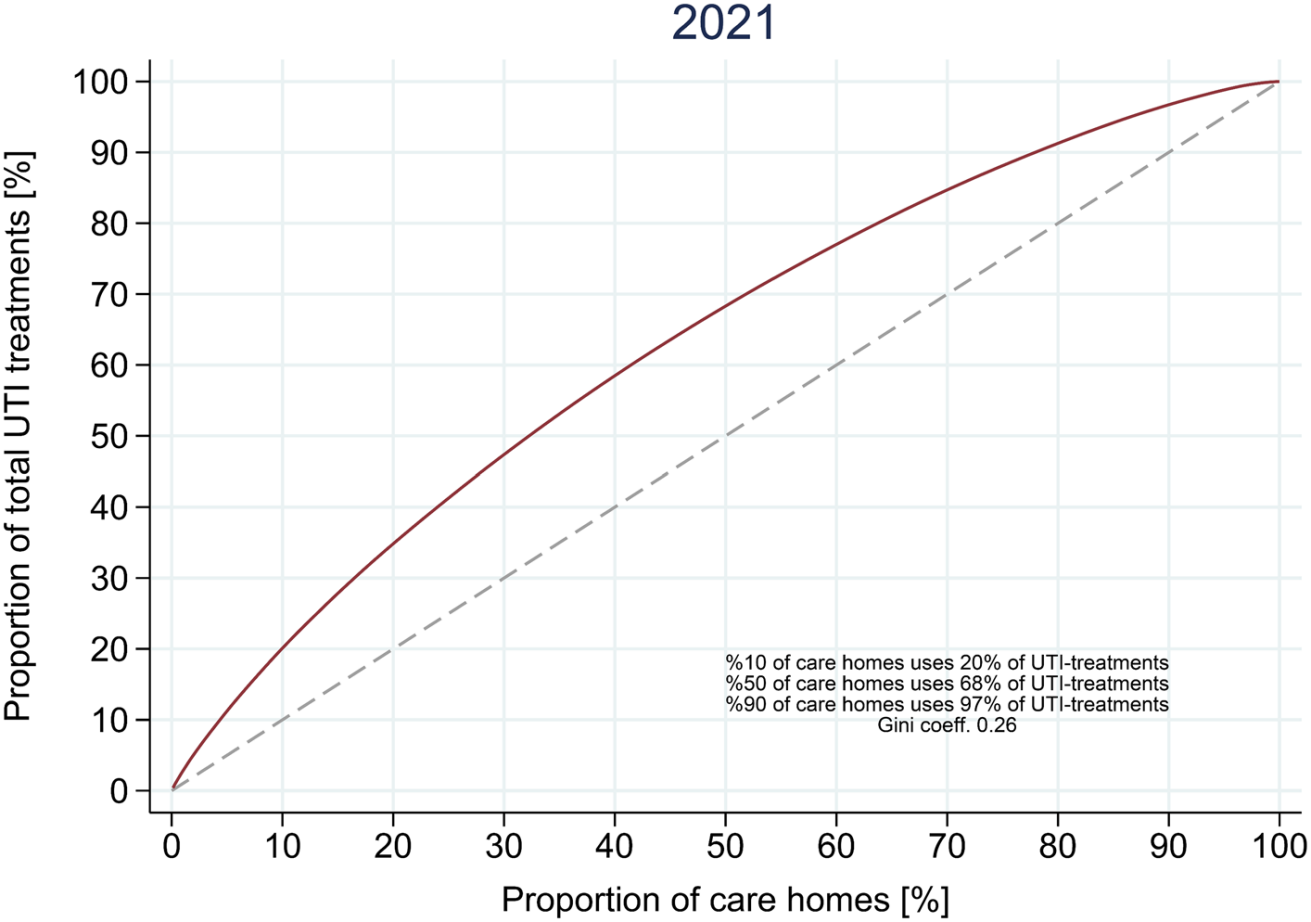
Differences in the use of urinary tract infections antibiotic treatments between different care homes during the year 2021 in Denmark. A treatment episode was defined as the filling of a prescription for a UTI antibiotic ≥ 15 days after a previous prescription

When looking at the level of the individual care home, we found considerable variation, with ten percent of care homes responsible for one fifth of all UTI treatments in 2021, and 50% were responsible for 68% of treatment episodes (Fig. 3). This skewness in use was consistent during the study period (Supplementary Fig. 5).

**Fig. 4:**
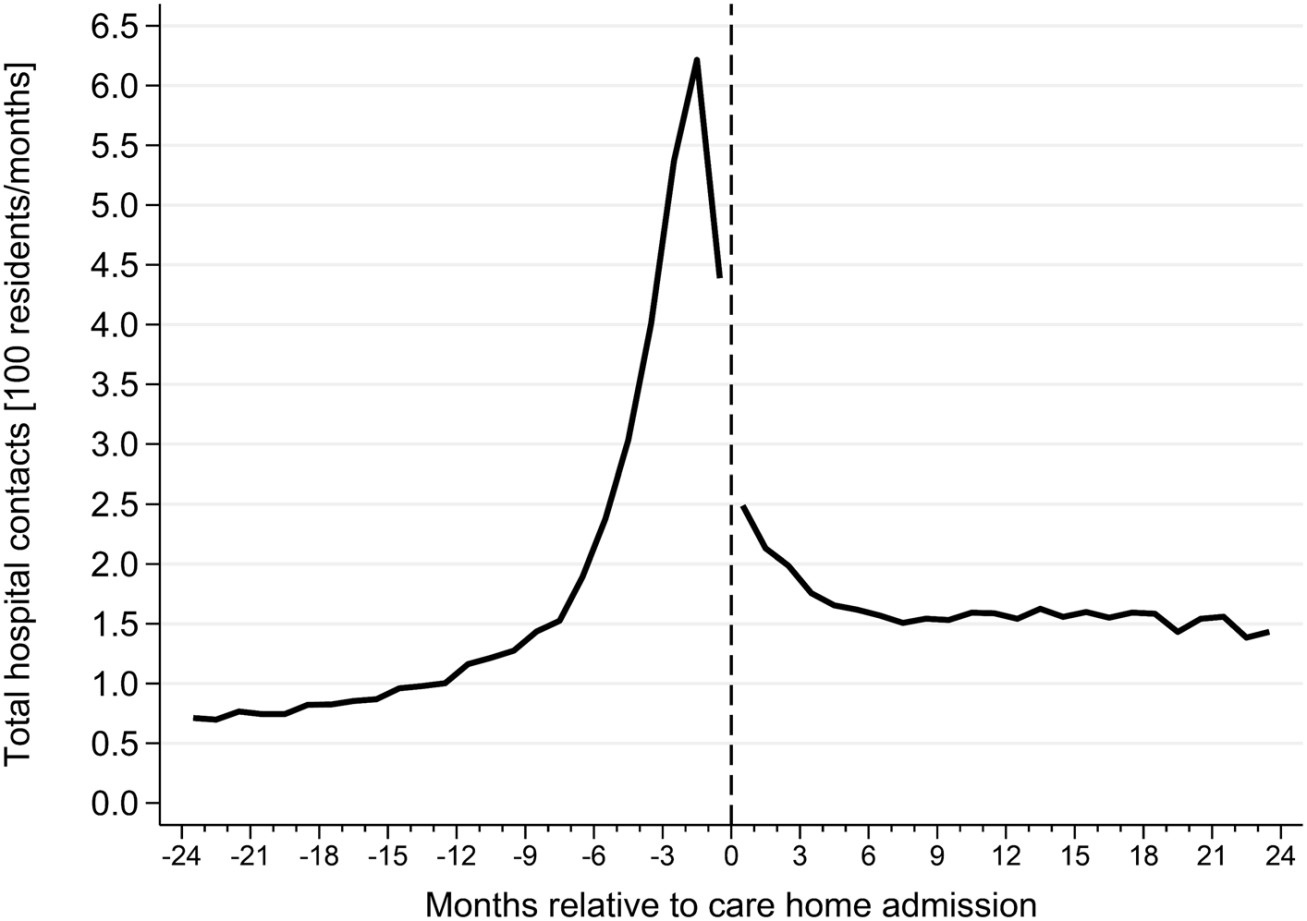
Total hospital contacts due to urinary tract infections related admissions in the two years leading up to and following care home admissions. UTI-associated contacts were defined by using the Danish National Patient Registry and the ICD-10 codes; cystitis (ICD-10: N30.X and N39.0); pyelonephritis (ICD-10: N10.X, N11.X and N12.X); observation due to suspected UTI (ICD-10: Z038A and Z038B); and urosepsis (ICD-10: A419B)

Hospital contacts related to UTIs peaked at two months prior to care home admission with six contacts per 100 residents per month (Fig. 4). However, contrary to the use of UTI antibiotics, where women used more medicine than men, men had more hospital UTI-related contacts following care home admission (Supplementary Fig. 6). The most prominent reasons for hospital contact were cystitis (N30.X, N39.0) and urosepsis (A419B), both among women and men (Supplementary Fig. 7). However, men were more often admitted due to urosepsis than women, whom were most often admitted due to cystitis. This trend persisted both during the year prior to and the year following care home admission. Among both sexes there was an increase in contacts due to urosepsis after moving into care homes from 7 to 11% and 18–25%, for women and men, respectively.

Lastly, we examined which types of physicians who most often prescribed UTI antibiotics. We found that the majority (81–97%) of all UTI antibiotics for care home residents was prescribed by practitioners in the primary healthcare sector (e.g., general practitioners or other specialists) (Supplementary Table 3). We also examined prescription patterns following care home admission and found that the percentage of UTI antibiotic prescriptions issued by general practitioners and other specialists increased with 3.6% during the two subsequent years (Supplementary Table 3). This increase was almost entirely made up for by a decrease of 3.7% by hospital physicians. Hospital physicians’ prescribing of UTI antibiotics decreased among all UTI antibiotic groups (− 1.0% to − 4.9%), while general practitioners’ prescribing increased for all UTI antibiotics (0.7–5.2%), with the highest increase seen with ciprofloxacin (5.2%) in the two years following care home admission.

## Discussion

We found that the use of UTI antibiotics increased in the six months prior to care home admission. In the two years following care home admission the use of UTI antibiotics decreased slightly but remained high compared to the time before care home admission. Hospital contacts related to UTIs also peaked around care home admission. Further, we found that, overall, women were more often treated with UTI antibiotics compared to men. However, men were more often admitted to hospital with a UTI-related diagnosis, which were often of a more serious/life threatening character, compared to women. Finally, we found an overall decrease in use patterns throughout 2016–2021, at both regional, institutional, and resident level, despite also showing variation at all levels.

A main strength of this study is the complete national cohort of all Danes admitted to nursing home in 2015 and onwards, with unambiguous linkage across Danish health registries. The study also has several limitations. First, we do not know the specific indication for why a certain antibiotic is prescribed, nor did we have access to biochemical or microbiological data. However, of the included antibiotics only ciprofloxacin is to some extent used for other non-UTI indications [37]. The final selection of UTI antibiotics included in this study was based on Danish Health Care Guidelines [25–30] and discussion with clinicians. As such, while overestimation of UTI antibiotic use is possible, only 8.2% of UTI antibiotic prescriptions were ciprofloxacin. Second, ICD-10 UTI-related diagnoses may not represent actual community-acquired UTI. A Danish validation study found that the positive predictive value for UTIs was 54% [38], thus supporting that misclassification of such contacts will have impacted our findings by leading to an overestimation of the number of hospital contacts related to UTIs. Thirdly, if patients are admitted due to UTIs in Danish emergency rooms, short course UTI antibiotics (e.g. around 3 days) can be provided directly in the emergency room without prescription. This use of UTI antibiotics is therefore not present in the data from the prescription registry, which only records prescriptions from community-pharmacies, possibly leading to an underestimation of the actual use.

A study examining use of UTI antibiotics among residents of long-term care facilities in over 20 different European countries similarly found that Denmark is among the countries with the highest proportion of use of UTI antibiotics among residents [13], comparable to Finland, Ireland, the Netherlands, and Wales. The wide variation in use of UTI antibiotics across regions, municipalities, care homes, and individual residents, may to some extend be due to the lack of a national guideline [25–29]. Interestingly, the fluctuations in use patterns seems to follow an overall national trend, although no single region consistently using more antibiotics than others. The potential explanations for this variation are unknown and could be related to factors such as changing local guideline or changes to clinical practice brought about by new local prescribers. Identifying drivers of low use of UTI antibiotics remain an area for further study. The increase in use of UTI antibiotics 6 months prior to care home admission may to some extent be related to presumed UTI diagnoses due to symptoms such as confusion and asymptomatic bacteriuria [16, 17]. These symptoms are commonly associated with the time period around care home admission, as a result of changes in health status [39]. Whether similar findings can explain the sustained high-level use of antibiotics following care home admission is unknown. These findings of high antibiotic use around time of care home admission and considerable variation at care home level is also reported in several studies examining the general use of antibiotics among care home residents [40–42].

### Clinical implication

A recent study testing a multifaceted antibiotic stewardship intervention on UTI prescriptions in frail older adults in Poland, the Netherlands, Norway, and Sweden found that their intervention reduced the number of prescriptions of UTI antibiotics safely [43]. These finding match a similar Danish cluster randomized trial [44]. Both intervention studies found that educating care home staff on UTIs and how to communicate with other healthcare professionals substantially lowered the use of UTI antibiotics and the risk of inappropriate prescribing [44–47]. This supports the importance of national guidelines on treatment regimen, and suggests that perhaps national efforts to increase the knowledge on UTIs and communication skills among care home staff may be effective in reducing the risk of inappropriate prescribing and use of UTI antibiotics [43]. Implementing national efforts may prove to be difficult, it may therefore be more efficient to initiate changes regionally. However, future studies on barriers for implementing such interventions is needed.

## Conclusion

We found that the use of urinary tract infection antibiotics is high among care home residents. The use increases prior to care home admission and remains at a stable high level during the two-year follow-up after care home admission. Further, we found variation in the use patterns at both regional, institutional, and residential level, despite also seeing an overall decrease in use throughout 2016 to 2021.

### Supplementary Information

Below is the link to the electronic supplementary material.

Supplementary file1 (DOCX 3954 KB)

## Data Availability

Individual level data cannot be shared by the authors owing to Danish data protection regulations. Deidentified data can be made available for authorised researchers after application to Forskerservice at the Danish Health Data Authority.
