# Supplementary material for: Use of antibiotics for urinary tract infections up to and after care home admission in Denmark: a nationwide study
Source: Eur Geriatr Med. 2024 May 2;15(3):797–805. doi: 10.1007/s41999-024-00976-1 (PMC11329397; doi:10.1007/s41999-024-00976-1)

SUPPLEMENTARY

INFORMATION

**Title: Use of antibiotics for urinary tract infections up to and after care home admission in Denmark: a nationwide study**

Emma Bjørk MScPharm, Rune Aabenhus MD PhD, Søren P. Larsen MScPharm, Jesper Ryg MD PhD, Daniel P. Henriksen MD PhD, Carina Lundby MScPharm PhD, Anton Pottegård MScPharm PhD DMSc

**Journal:** European Geriatric Medicine

**Correspondence:** Emma Bjørk, Clinical Pharmacology, Pharmacy and Environmental Medicine, University of Southern Denmark, JB Winsløwsvej 19, 2, DK-5000 Odense C, Denmark (ebjoerk@health.sdu.dk, 004521227795, @EBjoerk)

**Supplementary Table 1**

Table showing the 11 most common indications for use of UTI antibiotics in the two years prior to and following care home admission in Denmark in the period 2015-2021. Indication texts are divided into certain UTIs: bladder infection, urinary tract infection, prophylaxis for urinary tract infection, chronic urinary tract infection; Missing indications; Possible UTI-related: infection; and Others: diarrhea, colon infection, lung infection, skin- and soft tissue infection

| **Indication texts** | **Frequency, n (%)** | | | | |
| --- | --- | --- | --- | --- | --- |
|  | **Pivmecillinam** | **Trimethoprim** | **Nitrofurantoin** | **Sulfonamides** | **Ciprofloxacin** |
| **Certain UTIs**  Bladder infection, Urinary tract infection, Prophylaxis for urinary tract infection, Chronic urinary tract infection | 203,201 (97) | 59,271 (99) | 32,499 (99) | 39,501 (99) | 21,244 (71) |
| **Missing**  Missing | 1,413 (0.7) | 517 (0.9) | 425 (1.3) | 409 (1.0) | 390 (1.3) |
| **Possible UTI-related**  Infection | 4,290 (2.1) | 11 (0.02) | n < 5 | 5 (0.01) | 7,025 (24) |
| **Others**  Diarrhea, Colon infection, Lung infection, Skin- and soft tissue infection | 182 (0.1) | n < 5 | 0 (0) | n < 5 | 2,682 (9.0) |

**Supplementary Table 2**

Table showing proportion [%] of prescriptions following a given prescription within 15 days. Drugs are as follows: pivmecillinam (J01CA08), nitrofurantoin (J01XE01), trimethoprim (J01EA01), sulfonamide drugs (sulfamethizole (J01EB02), sulfamethoxazole/trimethoprim (J01EE01)), and ciprofloxacin (J01MA02)

| **First prescription** |  | **Prescription following within 15 days, n (%)** | | | | |  |
| --- | --- | --- | --- | --- | --- | --- | --- |
|  |  | Pivmecillinam | Trimethoprim | Sulfonamides | Ciprofloxacin | Nitrofurantoin | None |
| Pivmecillinam |  | 18,661 (51) | 5,437 (15) | 4,898 (13) | 4,149 (11) | 3,406 (9.3) | 181,223 (83) |
| Trimethoprim |  | 2,453 (24) | 6,207 (60) | 558 (5.4) | 589 (5.7) | 457 (4.5) | 59,706 (85) |
| Sulfonamides |  | 1,791 (33) | 880 (16) | 1,577 (29) | 615 (11) | 634 (12) | 35,478 (87) |
| Ciprofloxacin |  | 719 (21) | 413 (12) | 216 (6.5) | 1,696 (51) | 301 (9.0) | 29,743 (85) |
| Nitrofurantoin |  | 1,294 (24) | 496 (9.2) | 356 (6.6) | 485 (9.0) | 2,737 (51) | 31,257 (85) |

**Supplementary Table 3**

Table showing prescription patterns and differences in prescription patterns for UTI antibiotics from two years prior to and following care home admission, divided by type of provider (general practitioner, hospital physician, private practicing specialist)

| **Prescription patterns** | **General practitioner (%)** | **Hospital physician (%)** | **Private practicing specialist (%)** | **Unknown (%)** |
| --- | --- | --- | --- | --- |
| Pivmecillinam | 188,304 (86) | 17,748 (8.1) | 6,900 (3.1) | 6,628 (3.0) |
| Nitrofurantoin | 34,736 (94) | 1,184 (3.2) | 486 (1.3) | 581 (1.6) |
| Sulfonamides | 38,974 (94) | 843 (2.0) | 679 (1.6) | 828 (2.0) |
| Trimethoprim | 65,747 (93) | 2,469 (3.5) | 1,293 (1.8) | 1,156 (1.6) |
| Ciprofloxacin | 26,416 (79) | 5,704 (17) | 459 (1.4) | 772 (2.3) |
| **Difference in prescription patterns from two years prior to and two years following care home admission, %** | **General practitioner** | **Hospital physician** | **Private practicing specialist** | **Unknown** |
| All UTI drugs | +4.1 | -3.6 | -0.1 | -0.4 |
| Pivmecillinam | +4.9 | -4.7 | -0.1 | -0.1 |
| Nitrofurantoin | +2.4 | -1.5 | -0.1 | -0.9 |
| Sulfonamides | +1.4 | -1.0 | -0.1 | -0.3 |
| Trimethoprim | +3.2 | -2.2 | -0.1 | -0.9 |
| Ciprofloxacin | +6.3 | -4.8 | -0.4 | -1.0 |

**Supplementary Figure 1**Proportion of UTI treatment episodes, specified by different UTI antibiotics. Drugs are as follows: pivmecillinam (J01CA08), nitrofurantoin (J01XE01), trimethoprim (J01EA01), sulfonamide drugs (sulfamethizole (J01EB02), sulfamethoxazole/trimethoprim (J01EE01)), and ciprofloxacin (J01MA02). A treatment episode was defined as the filling of a prescription for a UTI antibiotic ≥ 15 days after a previous prescription.


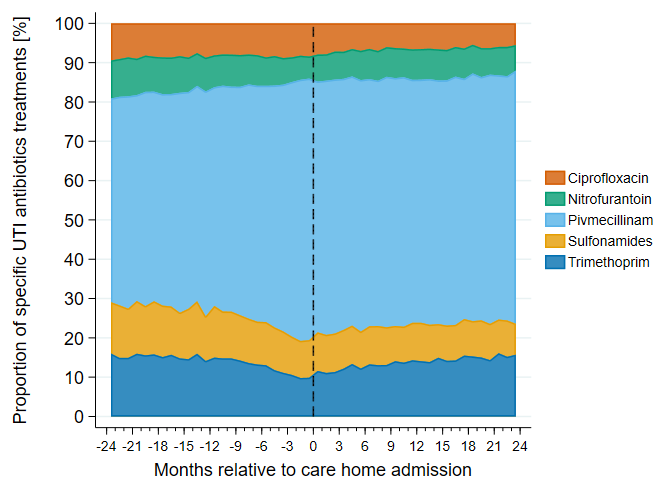


**Supplementary Figure 2**

Number of treatment episodes per month per 100 residents in the two years leading up to and following care home admission and specified by sex. A treatment episode was defined as the filling of a prescription for a UTI antibiotic ≥ 15 days after a previous prescription.

**Supplementary Figure 3**


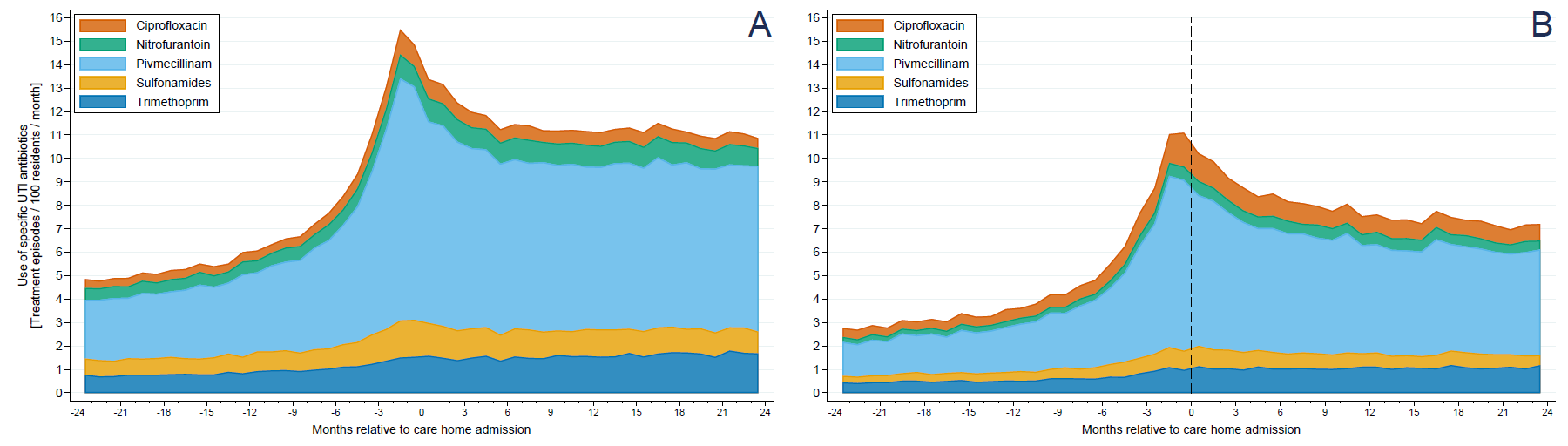
Number of treatment episodes among A) females and B) men per month per 100 residents in the two years leading up to and following care home admission and specified by type of antibiotic. A treatment episode was defined as the filling of a prescription for a UTI antibiotic ≥ 15 days after a previous prescription.

**Supplementary Figure 4**

Differences in the use of UTI antibiotics between the 98 municipalities of Denmark, during 2016 to 2021. Represented by average number of treatments per residents per municipality per year, standardized by sex and age to the overall population. A treatment episode was defined as the filling of a prescription for a UTI antibiotic ≥ 15 days after a previous prescription.

*
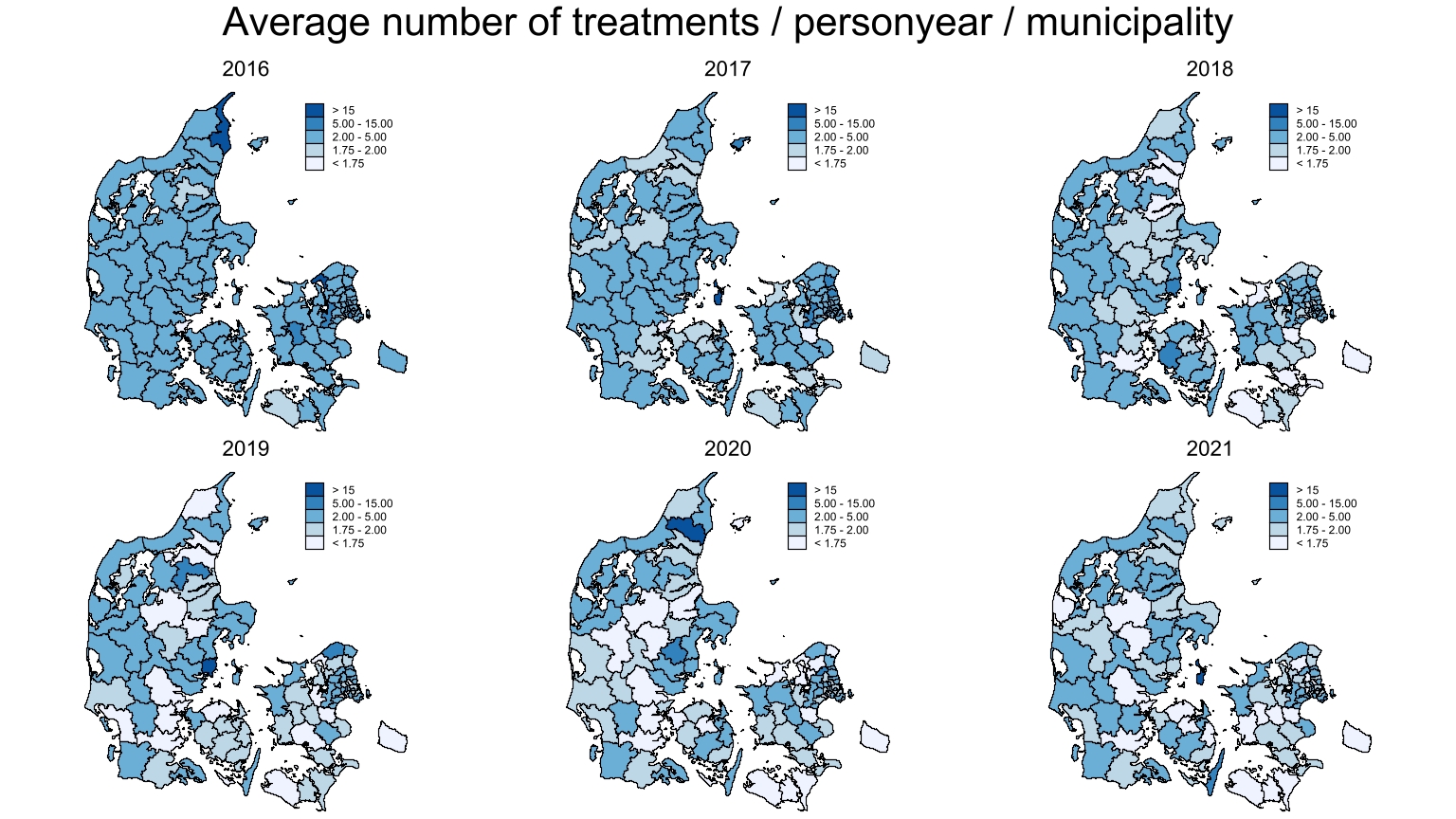
*

**Supplementary Figure 5**

Differences in the use of UTI antibiotic treatments between different care homes in the years 2016 to 2021 in Denmark. A treatment episode was defined as the filling of a prescription for a UTI antibiotic ≥ 15 days after a previous prescription.


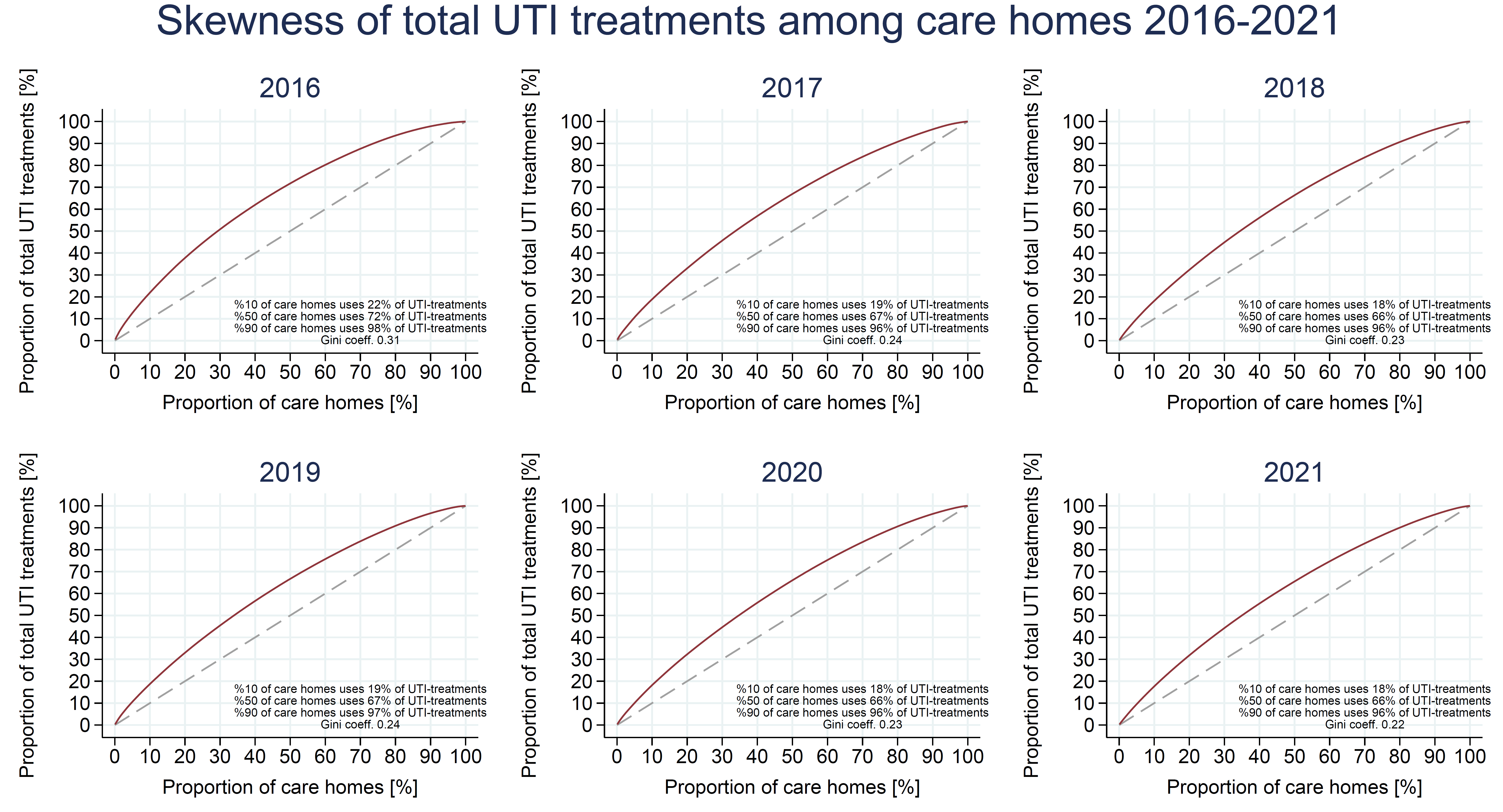


**Supplementary Figure 6**

Total hospital admissions due to urinary tract infection (UTI) related admissions in the two years leading up to and following care home admissions divided by sex. UTI related admissions were defined by using the Danish National Patient Registry and the ICD-10 codes; cystitis (ICD-10: N30.X and N39.0), pyelonephritis (ICD-10: N10.X, N11.X and N12.X), observation due to suspected UTI (ICD-10: Z038A and Z038B), and urosepsis (ICD-10: A419B).
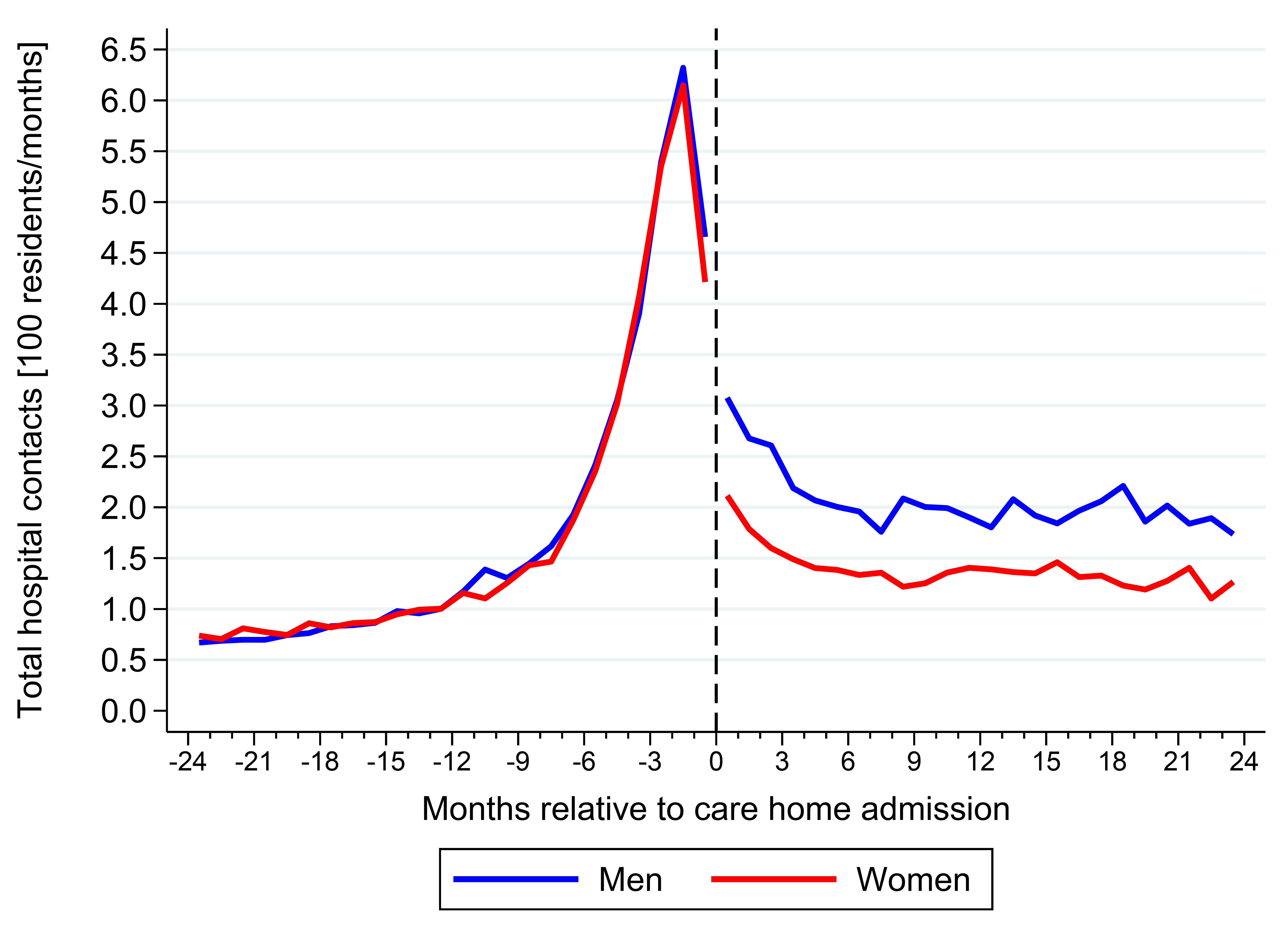


**Supplementary Figure 7**

Hospital contacts due to urinary tract infection (UTI) related admissions in the year prior to and following care home admissions divided by sex and specified by ICD-10 codes. UTI related admissions were defined by using the Danish National Patient Registry and the ICD-10 codes; cystitis (ICD-10: N30.X and N39.0), pyelonephritis (ICD-10: N10.X, N11.X and N12.X), observation due to suspected UTI (ICD-10: Z038A and Z038B), and urosepsis (ICD-10: A419B).


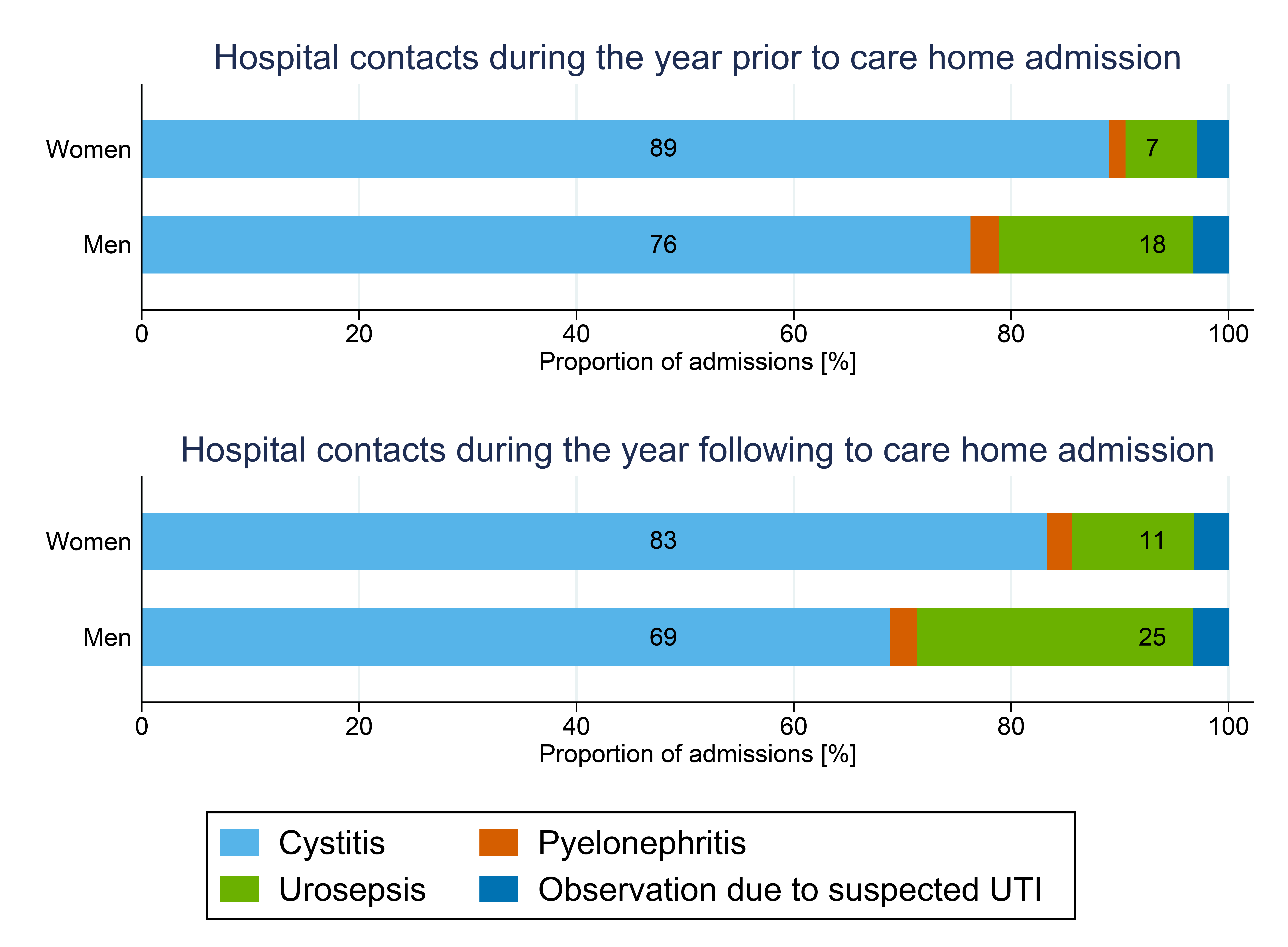

Supplement: Supplementary file 1 — Supplementary file1 (DOCX 3954 KB) [file 41999_2024_976_MOESM1_ESM.docx]
